# Supplementary material for: A reference DNA barcode library for Austrian amphibians and reptiles
Source: PLoS One. 2020 Mar 12;15(3):e0229353. doi: 10.1371/journal.pone.0229353 (PMC7067431; doi:10.1371/journal.pone.0229353)
Supplement: S2 Table — (DOCX) [file pone.0229353.s002.docx]

**Supplementary table 2:** Table containing all primer names, their nucleotide sequence and a reference to the original study used during the present research.

| **Primer name** | **Sequence** | **Source** |
| --- | --- | --- |
| Chmf4 | TYTCWACWAAYCAYAAAGAYATCGG | Che et al. 2012 |
| Chmr4 | ACY TCR GGR TGR CCRAAR AAT CA | Che et al. 2012 |
| Rep-COI-F | TNT TMT CAA CNA ACC ACA AAG A | Nagy et al. 2012 |
| Rep-COI-R | ACT TCT GGR TGK CCA AAR AAT CA | Nagy et al. 2012 |
| dgLCO-1490 | GGTCAACAAATCATAAAGAYATYGG | Meyer 2003 |
| dgHCO-2198 | TAAACTTCAGGGTGACCAAARAAYCA | Meyer 2003 |
| COI-C01 | TYTCWACWAAYCAYAAAGAYATTGG | Che et al. 2012 |
| COI-C02 | AYTCAACAAATCATAAAGATATTGG | Che et al. 2012 |
| COI-C03 | ACY TCY GGR TGA CCA AARAAY CA | Che et al. 2012 |
| COI-C04 | ACY TCR GGR TGA CCA AAA AAT CA | Che et al. 2012 |
| **C_VF1LFt1/ C_VR1LRt1** |  | Ivanova et al. 2007 |
| LepF1_t1 | GTAAAACGACGGCCAGTATTCAACCAATCATAAAGATATTGG | Hebert et al. 2004 |
| VF1_t1 | GTAAAACGACGGCCAGTTCTCAACCAACCACAAAGACATTGG | Ivanova et al. 2006 |
| VF1d_t1 | GTAAAACGACGGCCAGTTCTCAACCAACCACAARGAYATYGG | Ivanova et al. 2006 |
| VF1i_t1 | GTAAAACGACGGCCAGTTCTCAACCAACCAIAAIGAIATIGG | Ivanova et al. 2006 |
| LepRI_t1 | AGGAAACAGCTATGACTAAACTTCTGGATGTCCAAAAAATCA | Hebert et al. 2004 |
| VR1d_t1 | CAGGAAACAGCTATGACTAGACTTCTGGGTGGCCRAARAAYCA | Ivanova et al. 2006 |
| VR1_t1 | AGGAAACAGCTATGACTAGACTTCTGGGTGGCCAAAGAATCA | Ward et al. 2005 |
| VR1i_t1 | AGGAAACAGCTATGACTAGACTTCTGGGTGICCIAAIAAICA | Ivanova et al. 2006 |

References for Supplementary table 2:

Che J, Chen HM, Yang JX, Jin JQ, Jiang K, Yuan ZY, Murphy RW, Zhang YP. Universal COI primers for DNA barcoding amphibians. Mol. Ecol. Res. 2012;12(2): 247–258.

Hebert PDN, Penton EH, Burns JM, Janzen DH, Hallwachs W. Ten species in one: DNA barcoding reveals cryptic species in the neotropical skipper butterfly Astraptes fulgerator. Proc. Nati. Acad. Sci. U.S.A. 2004;101(41): 14812–14817.

Ivanova NV, Dewaard JR, Hebert PDN. An inexpensive, automation-friendly protocol for recovering high-quality DNA. Mol. Ecol. Notes. 2006;6(4): 998–1002.

Ivanova NV, Zemlak TS, Hanner RH, Hebert PDN. Universal primer cocktails for fish DNA barcoding. Mol. Ecol. Notes. 2007;7(4): 544–548.

Meyer CP. Molecular systematics of cowries (Gastropoda: Cypraeidae) and diversification patterns in the tropics. Biol. J. Linn. Soc. 2003;79(3): 401–459.

Nagy ZT, Sonet G, Glaw F, Vences M. First Large-Scale DNA Barcoding Assessment of Reptiles in the Biodiversity Hotspot of Madagascar, Based on Newly Designed COI Primers. PLoS ONE. 2012;7(3): e34506

Ward RD, Zemlak TS, Innes BH, Last PR, Hebert PDN. DNA barcoding Australia’s fish species. Phil. Trans. Royal Soc. B. 2005;360(1462): 1847–1857.
