# Supplementary material for: A reference DNA barcode library for Austrian amphibians and reptiles
Source: PLoS One. 2020 Mar 12;15(3):e0229353. doi: 10.1371/journal.pone.0229353 (PMC7067431; doi:10.1371/journal.pone.0229353)
Supplement: Attachment — Submitted filename: Response to Reviewers.docx [file pone.0229353.s003.docx]

Response to Reviewers

Journal Requirements:

1. We ensured that our manuscript meets the PLOS One style requirements, including file naming.

2a. We provided an amended Funding Statement declaring the commercial affiliation of one of the co-authors at the online forum.

2b. We provided an updated Competing Interest Statement at the online forum.

2c. We included both an updated Funding Statement and Competing Interest Statement in our cover letter and uploaded it onto the online forum.

General comments:

- How was morphological identification of species accomplished? This is particularly crucial for Pelophylax and Triturus. Please list the references / methods (and possibly the identifiers) by which / who the specimens were identified.

**Reply:** We listed the references used for determination of specimens of *Pelophylax* and *Triturus* and noted the link to geography, which aids in species assignment (as hybridization zones and distribution of ‘pure’ species are already known.

- Entire text: check writing: "DNA barcodes" vs. "DNA-barcodes"

**Reply:** We changed it to “DNA barcodes” throughout the whole manuscript.

- Is this the first publication of P. bergeri mtDNA in Austrian green frog samples? If yes, this should be highlighted more. If no, please cite the appropriate references.

Reply: Indeed, this is the first ever record of genetic signatures of *P. bergeri* recovered from Austria. At the reviewers’ suggestion, we emphasized this fact by adding “…the Italian water frog *Pelophylax bergeri* in Western Austria for the first time.” in the abstract and the discussion and “…the first ever recovered genetic signatures…” in the results section. However, since we found it only in a single individual and since we cannot be sure whether this animal could have been transported or introduced by humans or migrated to Austria naturally, we opted to present this discovery with caution and reservation.

Specific:

l. 65: Change "biodiversity assessments" to "studies on biodiversity". This facilitates understanding the entire sentence.

**Reply:** We agree and changed it to “studies on biodiversity”.

l. 68: Delete "singule".

**Reply:** Done.

l. 81: Change "V. ursinii rakosiensisi" to "V. ursinii rakosiensis". Change order: "the in Austria putatively extinct V. ursinii rakosiensisi" to "the putatively extinct in Austria V. ursinii rakosiensis".

**Reply:** Done.

l. 119: Change "L. helvetica" to "L. helveticus".

**Reply:** Done.

l. 142: Remove period / full stop "." after "BOLD".

**Reply:** Done.

l. 145 onwards: "Austrian Pelophylax formed three clades…" Were species names assigned based on morphological identification of specimens or on DNA barcodes? I assume this is morphological ID, but please specify.

**Reply:** Specimens were first up determined based on their morphological appearance, following best practice guidelines for the generation of DNA-barcodes. To clarify, we added “…, determined based on their morphology,…”.

l. 168: "Imax": Explain the abbreviation.

**Reply:** Done. We included “maximum intraspecific distance (I_max_)” as an explanation for the abbreviation at first mentioning.

l. 173, Table 1: Change "Natrix tesselate" to "Natrix tesselata".

**Reply:** Done.

l. 197: See comment to l. 81.

**Reply:** Done like for line 81.

l. 338: Change "personal" to "staff".

**Reply:** Done.

Check reference list for correct format. E.g., l. 356: change "2003; 12: 5–12." to "2003;12: 5–12."

**Reply:** Done. We changed line 356 and checked the rest of the references and made the necessary corrections if needed.
